# Current and Future Perspectives of the Use of Organoids in Radiobiology

**DOI:** 10.3390/cells9122649

**Published:** 2020-12-09

**Authors:** Peter W. Nagle, Robert P. Coppes

**Affiliations:** 1 Cancer Research UK Edinburgh Centre, MRC Institute of Genetics and Molecular Medicine, University of Edinburgh, Edinburgh EH4 2XR, UK; 2 Department of Biomedical Sciences of Cells & Systems, University Medical Center Groningen, University of Groningen, Groningen, 9700 RB Groningen, The Netherlands; r.p.coppes@umcg.nl; 3 Department of Radiation Oncology, University Medical Center Groningen, University of Groningen, Groningen, 9700 RB Groningen, The Netherlands

**Keywords:** Radiation, radiobiology, stem/progenitor cells, organoids

## Abstract

The majority of cancer patients will be treated with radiotherapy, either alone or together with chemotherapy and/or surgery. Optimising the balance between tumour control and the probability of normal tissue side effects is the primary goal of radiation treatment. Therefore, it is imperative to understand the effects that irradiation will have on both normal and cancer tissue. The more classical lab models of immortal cell lines and in vivo animal models have been fundamental to radiobiological studies to date. However, each of these comes with their own limitations and new complementary models are required to fill the gaps left by these traditional models. In this review, we discuss how organoids, three-dimensional tissue-resembling structures derived from tissue-resident, embryonic or induced pluripotent stem cells, overcome the limitations of these models and thus have a growing importance in the field of radiation biology research. The roles of organoids in understanding radiation-induced tissue responses and in moving towards precision medicine are examined. Finally, the limitations of organoids in radiobiology and the steps being made to overcome these limitations are considered.

## 1. Introduction—Optimising the Therapeutic Window of Radiation Treatment

**Figure 1:**
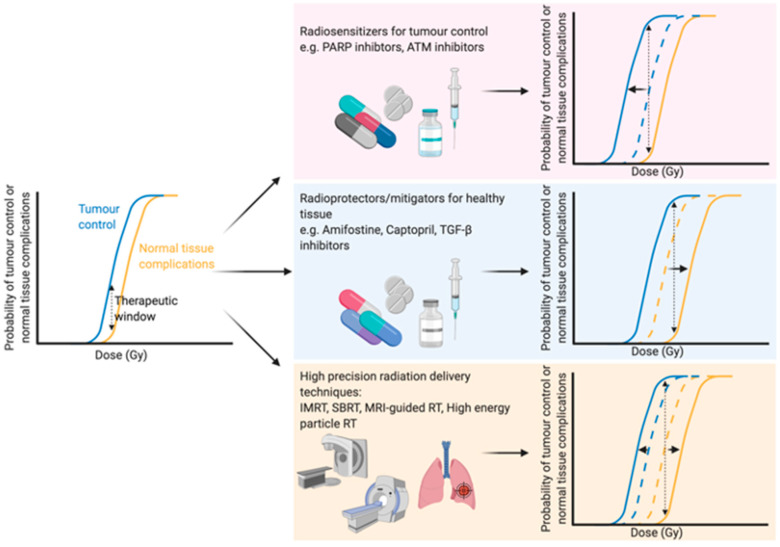
Optimising the therapeutic window of radiotherapy. The therapeutic window describes the balance between the probability of tumour control (blue line) and normal tissue complications (yellow line). There are three main rationales behind broadening the therapeutic window in radiation treatment: (1) Increasing tumour sensitivity using radiosensitisers, reducing the dose required for tumour kill (blue line shifts to the left), (2) protecting normal tissue using radioprotectors or mitigators, thus increasing the tolerable dose of normal tissue (shifting the yellow line to the right) or (3) high precision dose delivery which can reduce the volume of co-irradiated normal tissue (effectively shifting the yellow line to the right) while in the case of charged particles an increased relative biological effectiveness reduces the dose required for tumour control. Abbreviations: PARP; poly-ADP ribose polymerase, ATM; ataxia telangiectasia mutated, TGF-β; transforming growth factor beta, IMRT; intensity-modulated radiation therapy, SBRT; stereotactic body radiation therapy, MRI; magnetic resonance imaging. Created with BioRender.com.

With an ever-aging population the number of people diagnosed with cancer is constantly growing [1]. Therefore, there is an even greater onus on the need to develop both current and new methods to enhance the efficacy of cancer treatments. Traditional cancer treatments, such as radiotherapy, chemotherapy and surgery, are still the most common modalities, but newer treatments such as immunotherapy are becoming more and more prevalent. Radiotherapy (either alone or in combination with surgery and/or chemotherapy) is used to treat over half of all cancer patients, with a curative intent in the majority of these cases [2,3]. Furthermore, the number of patients undergoing radiotherapy is predicted to increase even further due to an aging and growing population, as well as rapid technological advances in radiotherapy delivery practices [4]. The primary goal of radiotherapy, as with all other forms of cancer treatment, is to maximise the therapeutic window. The therapeutic window describes the balance between the probability of increasing tumour cell kill while minimising the probability of normal tissue complications. This can be achieved by using drugs which target the intrinsic vulnerabilities of a tumour to make it more susceptible than healthy tissue, or alternatively by physically targeting the tumour with greater accuracy and minimising the co-irradiated normal healthy tissue (Figure 1).

The development of high precision means of dose delivery, such as intensity modulated radiation therapy [5], stereotactic radiation therapy [6] and charged particle radiotherapy [7], have allowed for substantial reductions in co-irradiated normal tissue during therapy. These strategies enable better sparing of crucial organs [8] or sub-regions [9] within organs during treatment or dose escalation to the tumour. Furthermore, real-time advanced imaging, such as magnetic resonance imaging (MRI), during radiation therapy has been suggested as a means to further optimise the delivery of radiation to the target tumour with an increased sparing of the surrounding healthy tissue. Initial in vitro studies showed no changes in survival in response to X-rays when a magnetic field of 1.5 T was applied [10]. Indeed, combining X-ray therapy with MRI-guidance has been successfully applied in clinical practice to increase the accuracy of dose delivery and thus spare a greater proportion of healthy tissue [11]. Particle therapies, such as proton therapies, can modulate the dose to encompass the whole tumour in a so-called “spread-out Bragg peak” with a minimised entrance dose and negligible exit dose, sparing healthy tissue [12,13]. Furthermore, MRI-guided proton therapy has also been proposed [14,15] and early in vitro findings suggest that a magnetic field perpendicular to the radiation beam has no effect on the radiobiological effectiveness of the dose [16], while a magnetic field longitudinal to the beam slightly changes the effectiveness [17], emphasising the potential of such advances in a clinical setting. Further advances in radiation delivery include FLASH radiotherapy, which delivers ultra-high dose rates of ionising radiation which are believed to reduce normal tissue complications compared to conventional dose rates [18], although the therapeutic window of FLASH therapy still needs to be addressed [19].

All of these technological advances in the field of radiation beam delivery have significantly reduced the amount of co-irradiated healthy tissue during radiation treatment; however, none of these developments can completely eliminate dose to the surrounding tissue. Therefore, it is still necessary to develop in vivo and in vitro models to improve understanding of the mechanisms involved to better protect and/or regenerate normal tissue or to target intrinsic vulnerabilities of a tumour to enhance radiotherapy efficacy. These models should also take the therapeutic window into account as there is often an overlap between these mechanisms in both normal tissue and tumours, a feature which is regretfully often overlooked. Here we discuss one such in vitro model which could potentially allow the comparison of normal tissue and tumour responses at a patient-specific level, organoids, and the ever-growing role it has in radiobiological studies. We examine the strength of organoids in mechanistic studies in both normal and diseased tissue, but also examine the prospects of organoids in a more personalised medicine approach for patients. Finally, we discuss (potential) developments within the field of organoid research that could further benefit the radiobiology world.

## 2. The Need for New Models in Radiobiology

**Figure 2:**
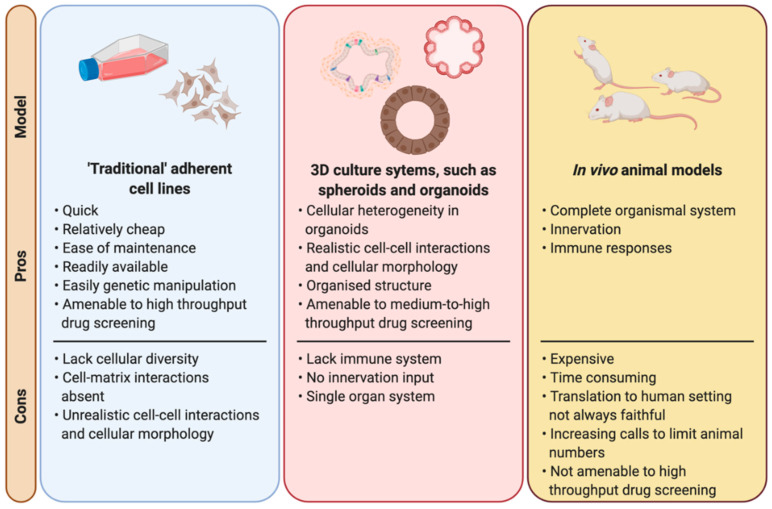
Laboratory models used in radiation biology. A comparison between the main laboratory-based models used in radiobiological studies highlighting the pros and cons of each model. Created with BioRender.com.

Since the beginning of the use of radiation treatment for cancer, radiobiology has made use of many different models to understand the molecular pathways triggered by radiation and to determine the consequences of radiation at a cellular, organ and system level in order to maximise the therapeutic window (Figure 2). Traditional cell cultures have been fundamental to radiobiology research, with many different techniques and findings crucial to other fields of biology, such as the development of the clonogenic survival assay [20,21]. Moreover, cell lines are highly amenable to high throughput drug screens, which in the field of radiobiology facilitates the efficient screening of large panels of potential radiosensitising agents over a radiation dose range [22]. However, cell lines cultured in two dimensions lack many features that are crucial to the overall response and survival of organisms following irradiation, such as cellular heterogeneity, cell–matrix interactions, “real” cell–cell interactions, a correct morphology and polarity, and functional relevance such as cytokine secretion [23,24]. Therefore, while they are invaluable, findings in cell lines often overstate findings, such as survival, compared to in vivo [25,26] and must therefore be treated with caution when translating to a more clinical patient setting.

Another model which has always been considered as a cornerstone for radiobiological research are in vivo animal models. Obviously animal models overcome many of the limitations of cell lines mentioned earlier, but they come with their own drawbacks, such as translatability to human settings, they are time consuming and expensive. Animal models are the most complete model available to researchers with the complete diversity of cell types and molecular interactions on an organismal level, as opposed to being constricted simply to a single cell type of a particular tissue when working with cell lines. Animal models are amenable to genetic manipulation and genetically modified animals offer the opportunity to study the impact of disease specific mutations, which in radiobiology allows researchers to study the effects on radio-resistance or -sensitivity of particular cancer associated mutations, such as *p53* [27,28] or *Atm* [29,30]. Furthermore, in vitro and in silico findings should always be confirmed in vivo as the final step prior to human translation, and therefore animal models will remain crucial to biomedical and radiobiological research. However, with an increasing growing pressure on researchers to limit (or even eradicate) the use of animals in research [31], it is necessary to find and implement alternative models in the search for treatments to a wide variety of diseases, not just cancer.

Organoids, three-dimensional in vitro structures derived from induced pluripotent stem cells, embryonic stem cells or tissue specific resident stem/progenitor cells [32,33], offer a “steppingstone” between more traditional in vitro cell lines and in vivo animal models. Organoids are self-assembling structures which resemble the tissue of origin [32,33,34]. They contain multiple cell types [32], overcoming the lack of cellular diversity of cell lines, although vasculature and endothelial cells are generally absent from these cultures. Distinct nomenclature has been proposed in some fields to distinguish between different 3D in vitro cultures, such as the suggested nomenclature differences between “enteroids”, “colonoids” and “organoids” in the gastrointestinal field [35]. Furthermore, the term “tumouroids” is frequently used for tumour-derived organoids (or tumour-like organoids). Therefore, it should be noted that here we use the term “organoids” to encompass all self-organising 3D cellular structures derived from embryonic stem cells, induced pluripotent stem cells or tissue-resident stem/progenitor cells which contain multiple different cell types found within the tissue of origin. This is based on the definitions proposed by Lancaster and Knoblich (2014) [32] and Clevers (2016) [33].

As they are cultured in three dimensions, the cellular interactions and morphology become more “realistic” allowing for endpoint readouts which more closely resemble clinical observations. Furthermore, many organoid cultures have been shown to secrete functional enzymes under the right conditions [36], while transplantation of cultured organoids into murine models has been shown to rescue injured phenotypes [37]. Following radiation treatment, normal tissue stem cells are crucial to tissue regeneration. Conversely, cancer stem cells have increased radioresistance, repopulate tumours and are more prone to metastasize [38]. Therefore, it is important to be able to assess stem cell responses and the dynamics of those responses within the cellular heterogeneity (consisting of stem cells, progenitors and differentiated cells) of the tissue of origin. As they are derived from stem/progenitor cells, organoids can be used as a readout for such cells in an environment encompassing such heterogeneity [26]. Organoids are crucial to the studies of the mechanistic sequalae to irradiation, but also have an increasing role and potential in a more personalised approach to determining individual patient treatments. However, when designing experiments using organoids, researchers should always consider the question on hand when deciding which model (tissue-derived organoids, embryonic stem cell-derived or induced pluripotent stem cell-derived) to be used. For example, in cancer studies using organoids, pluripotent stem cell-derived and CRISPR-edited normal tissue-derived organoids can mimic germline mutations and thus allow accurate assessment of specific mutations in oncogenesis [39]. However, for treatment response studies, patient-derived organoids may represent a more suitable model, as they can encompass the true complexity of the disease, such microsatellite and chromosomal instabilities [40,41].

## 3. Organoids and Regeneration of Radiation-Induced Damaged Tissue

Since the identification of Lgr5 as a marker for intestinal stem cells [42], one of the most studied and established organoid models are the gastrointestinal “mini-gut” organoids. Originally established from mouse small intestinal stem cells [43], organoid “mini-gut” models have subsequently been established from human stem cells [44], as well as from various different locations along the gastrointestinal tract, including stomach [45], colon [44] and oesophagus [46]. Furthermore, pluripotent stem cells have been utilised to successfully generate intestinal [47] and oesophageal [48] organoid cultures. These models have opened novel avenues of study for intestinal development, cancer progression [49] and other diseases, such cystic fibrosis [50].

While there have been only a limited number of studies using organoids to investigate radiation-induced gastrointestinal injury, some recent studies have used organoids to complement and reinforce important insights from in vivo mouse studies [51,52,53]. Wang et al. [51] demonstrated using intestinal crypt organoids that selective inhibition of radiation-induced p53-mediated apoptosis using CHIR99021, an inhibitor of glycogen synthase kinase-3 (GSK-3), can protect intestinal stem cells against radiation due to an increased survival of Lgr5+ cells. This was recapitulated in vivo, indicating a pivotal role for p53 post-translational modifications in intestinal stem cell responses to irradiation [51]. More recently, using intestinal organoids from mouse jejunum and human colon, Bhanja et al. [52] revealed the potential of BCN057, an anti-neoplastic small molecular agent, to mitigate radiation-induced gastrointestinal syndrome in normal tissue. Interestingly, BCN057 did not have a radiomitigative effect in tumour-derived organoids with these findings again mimicking in vivo findings. The same group also investigated the potential of repurposing auranofin, an anti-rheumatoid drug containing gold, as a radioprotective agent against intestinal injury [53]. In both in vivo mice and ex vivo human colon organoids treatment with auranofin significantly reduced the toxicity of radiation [53]. Furthermore, Martin et al. [54] recently demonstrated that the profile of the Lgr5+ stem cell population of the large and small intestines following irradiation of organoids could act as a marker for predicting the sensitivity of these organs to radiation. The authors validated their approach using organoids with a well-established in vivo microcolony assay which quantifies the number of regenerating crypts per small intestinal circumference [54,55]. This assay is regarded as a benchmark assay for establishing the radiosensitivity of intestinal stem cell survival and highlights the potential of intestinal organoids to predict radiation responses [54]. These studies demonstrate the strength of “mini-gut” organoids as a model for radiation studies of the gastrointestinal tract and also the opportunities for radiobiological studies in other organoid systems, particularly in tissues which lack accurate in vitro models for radiobiological studies.

Radiotherapy is used to treat the majority of head and neck cancer patients, either alone or in combination with surgery and/or chemotherapy [56]. Frequently, irradiation of head and neck tumours leads to the unavoidable co-irradiation of salivary glands, with almost half of head and neck cancer patients subsequently suffering from radiation-induced xerostomia due to hyposalivation. This drastically impacts on the quality of life of patients due to impaired chewing, swallowing, speaking and an increased risk of oral infections [57]. In vivo studies using rats have shown that sparing a region of the salivary gland which contains a high density of tissue specific stem/progenitor cells has been shown to reduce the effects of salivary gland irradiation [9]. Therapeutic options are available to stimulate salivary gland flow post-irradiation but are limited in their effectiveness [57]. Therefore, a need for a more long-term strategy for salivary gland regeneration following radiotherapy remains [58]. While in vivo animal models have provided a wealth of knowledge as to the mechanisms behind salivary gland regeneration following injury, including radiation-induced damage [59,60,61,62,63,64], there is a limited number of in vitro systems to accurately study salivary glands following irradiation. Thus there is a growing niche for new models such as organotypic slice cultures [65] and organoids in the area of salivary gland radiation research.

Recently, our group has established protocols for the isolation and expansion of both murine [66] and human [67] submandibular salivary gland stem/progenitor cells. Using these protocols, we have shown that transplantation of enriched murine or human stem/progenitor cell populations improved functional readouts of irradiated mice salivary glands [37,67,68]. However, this effect may not only be directly from the expansion of the stem/progenitor cells in the transplanted tissue, but also due to paracrine effects of the transplanted cells acting on the recipient tissue [67]. Another recent study by Tanaka et al. has demonstrated the ability to derive salivary gland stem cells from embryonic stem cells [69]. Upon transplantation into parotid gland-defective mice, the induced salivary gland cells (transplanted either alone or together with mesenchymal cells) were capable of generating mature salivary gland tissue. The newly generated tissue was also shown to be functional as demonstrated by an increased saliva secretion in transplanted mice [69]. Combined, these studies hold significant preclinical promise for studying the mechanisms behind salivary gland regeneration and amelioration of salivary gland damage, both irradiation and non-irradiation induced damage [67,69]. However, the translation of any embryonic stem cell derived treatment [69] to a clinical application is always likely to be hindered by ethical concerns [70] and safety concerns regarding tumorigenicity [71].

Our models have been successfully utilised to study the survival responses of salivary gland stem/progenitor cells [26]. The salivary gland stem/progenitor organoids demonstrated a disproportionate sensitivity to low dose of radiation which was recapitulated in a functional low dose sensitivity in vivo [72]. While low dose hypersensitivity is not a new phenomenon [73,74], this was the first study to show the relevance of this phenomenon in stem/progenitor cells, with a potential clinical relevance. Furthermore, we have recently developed a protocol for the culturing of parotid salivary gland organoids and demonstrated that parotid gland stem cells display a similar radiosensitivity as those of submandibular salivary glands [75]. Importantly, as organoids are derived from stem/progenitor cell populations, they allow for the study of a more stem/progenitor specific response. As stem/progenitor cells play a prominent role in tissue regeneration following irradiation, models which allow for the understanding of these cells are crucial to protecting these tissues.

Another tissue in which the use of radiation is highly limited due to radiation-induced toxicity is the liver. Along with lung, breast, colorectal and pancreatic cancers, liver cancer deaths are one of the highest of all cancer-related deaths each year [76], while the prognosis is extremely poor due to limited treatment options [77]. The use of radiation treatment for liver cancer is severely hindered by the development of radiation-induced liver disease [78], a consequence which can also impede the utilisation of radiotherapy for other abdominal tumours in proximity to the liver, such as gastrointestinal cancers [79]. Much of what is known regarding radiation-induced liver disease is from retrospective clinical studies [79], as current lab models for studying it are limited with in vitro studies generally limited to cell lines lacking cellular heterogeneity and functionality. The recently developed models of both mouse [80] and human [81] derived liver organoid cultures from tissue resident stem cells, as well as pluripotent stem cell-derived liver cultures [82,83,84,85], may represent an ideal model for studying radiation-induced liver disease in the future. These models display cellular, functional activity and have structural organisation, while they have been successfully utilised to study genetic liver disorders mimicking the clinical pathology [81] and drug-induced liver injury [86]. Understanding the mechanisms of radiation-induced liver disease may eventually allow for increased treatment options for liver cancers.

## 4. A Platform for Treatment Response Studies; Moving towards Personalised Treatment?

The concept of precision treatments has been of growing interest in many fields of research in recent years, particularly oncology, as there is a wide variability of patient responses to standard “one size fits all” treatment regimens. In some cases, genetic factors which can be specifically targeted in a “personalised” manner are already known, for example non-small cell lung cancer patients with an activating mutation in tyrosine kinase are particularly sensitive to treatment with tyrosine kinases inhibitors such as gefitinib [87]. However, for other cancers, such as oesophageal cancers and locally advanced rectal cancers, there are currently no accurate predictors of patient responses to treatment. The standard of care for oesophageal cancer consists of neo-adjuvant chemoradiotherapy followed by surgery, with a complete pathological response observed in approximately a quarter at the time of surgery but no response in approximately one fifth of patients [88,89]. Similarly, for neoadjuvant chemoradiotherapy treatment of colorectal cancer while approximately one fifth of patients show a complete pathological response, almost 40% of patients show no benefit to the treatment [90]. In both cancers, patients would clearly benefit from more robust pre-treatment predictive models.

Therefore, there has been a concentrated effort in the field of organoids to establish reliable predictors of colorectal cancer treatment response to both chemotherapy alone [91,92] and neoadjuvant chemoradiotherapy [93,94]. Van de Wetering et al. [91] established colorectal cancer organoids, alongside paired healthy tissue, and demonstrated that the organoids recapitulated the genetic profiles and mutational spectra of the tumours of origin. Furthermore, by performing screening of 83 compounds, including both clinically used drugs and experimental compounds, the authors showed that the organoids facilitated the high-content drug screening [91], which could facilitate precision treatments in the future. Interestingly, a later study by Ooft et al. [92] investigating treatment response of metastatic colorectal cancer using organoids, was able to predict accuracy of irinotecan monotherapy and 5-flurouracil/irinotecan dual therapy, with 80% and 83.3% respectively. While greater accuracy is required to implement predictive models in a clinical setting, these studies show the developing potential of organoids in precision medicine. Furthermore, in recent years, there has been an increasing number of studies aimed at identifying and repurposing already available drugs as radiosensitisers [95,96,97,98]. Drugs which can be repurposed offer cheaper and quicker alternatives to developing new drugs from scratch, while many of the adverse side effects are already known [99]. The possibilities to quickly and accurately screen drugs, as shown in the studies of van de Wetering et al. [91] and Ooft et al. [92], in cancer organoids will greatly increase the possibilities in precision medicine and further benefit the search for potentiators of radiation therapy.

Indeed, recent studies by Ganesh et al. [93] and Yao et al. [94] have focussed on rectal cancer organoids for predicting patient responses to neoadjuvant chemoradiotherapy (Table 1 summarises the different cancer organoids that have been used in studies of radiation responses). Both studies further consolidated other evidence that rectal cancer organoids faithfully recapitulate the tumours of origin, performing histopathological and mutational comparisons between the two [93,94]. Moreover, Ganesh et al. showed that upon xenotransplantation of the organoids into mice they were found to metastasise to the same locations as the original tumours. Importantly, upon treating the organoids with chemotherapeutic drugs (such as 5-Flurouracil and oxaliplatin) heterogeneous treatment responses correlated with the clinical progression-free survival of patients. Interestingly, organoids which displayed resistance to radiation were derived from patients who either were resistant to therapy or showed disease recurrence following treatment [93]. Yao et al. [94] also correlated the therapeutic clinical outcomes to the standard neoadjuvant chemoradiotherapy with the organoid outcomes following treatment 5-Flurouracil, irinotecan or radiation. In sixty-eight out of the 80 patient-derived organoid lines generated, at least one of the three treatment courses was found to be predictive of the patient’s tumour regression score after surgery [94]. Furthermore, in a recent study, Pasch et al. established patient-derived cancer organoids and were prospectively able to predict the treatment response of a patient with metastatic colon cancer [100]. These studies combined with the works of van de Wetering et al. and Ooft et al. provide a significant step towards a model for patient-specific response prediction.

A recent study also established an organoid model for metastatic gastrointestinal cancers which were histologically, genetically and molecularly similar to the tumour of origin [104]. Following drug treatment of the organoids, the outcomes were compared with the clinical outcomes of the patients enrolled in Phase I/II clinical trials and were found to closely mimic the clinical outcomes of the patients [104]. Moreover, the study successfully identified differential inter- and intra-patient responses to common chemotherapeutic agents for gastrointestinal cancer treatment [104]. This study represents an important advance for organoids in the field of personalised precision medicine.

As mentioned above, currently the ability to predict patient responses to chemoradiotherapy for oesophageal cancer is also extremely limited. Great strides are being made towards the optimisation of imaging techniques for predicting treatment outcomes for oesophageal cancer treatment [89,105,106,107]; however, there is still no means to accurately predict patient outcomes. Recent advances in the culturing of oesophageal adenocarcinoma organoids have established new models to study the development and heterogeneity of the disease [108]. The established patient-derived oesophageal adenocarcinoma organoids shared histopathological features with patient-matched tumour samples and genetic mutations were conserved at a patient-specific level [108]. They further showed a loss of cellular polarity, which is often considered a hallmark of cancer. Drug screening in the organoids revealed a highly diverse range of responses, which tallies with the difficulties in predicting patient responses. However, the diversity of the responses remained throughout passaging, indicating the stability of the model through time [108]. Unfortunately, the findings of this study were somewhat limited due to a low success rate of establishing organoids (organoids were established from only 10 out of 32 patients). Reasons for a low success yield included failure to initiate culture, infection, fibroblast overgrowth, and arrested growth [108], while others also working on developing oesophageal adenocarcinoma organoids have recently identified the presence of Barrett’s epithelium as another potential contamination source in culture [109]. These new models will be essential to opening new avenues for testing new drugs and treatment regimens for oesophageal adenocarcinomas. Furthermore, as mentioned above, radiotherapy is an also important arm of treatment for other cancers in the head and neck region. Recently established protocols for generating organoids from oral mucosa and head and neck squamous cell carcinomas may facilitate a more personalised treatment planning for more tumours in this region [101]. Comparisons of the responses of tumour organoids with matched normal tissue organoids may even allow for studies of the therapeutic window on a personalised scale.

Glioblastoma is a highly aggressive brain tumour with an extremely poor prognosis for patients for whom radiotherapy is an integral arm of treatment [110]. This remains the case even with significant advances in the understanding of glioblastoma development, cellular heterogeneity within the tumour, and the role of cancer stem cells play in this [111,112,113]. Many of the models used for studying glioblastoma utilise adherent monolayers which, although they have been highly revealing of the mechanisms of glioma stem cell resistance [114], have thus far not been representative of the tumour microenvironment or levels of therapeutic resistance of glioblastoma seen in vivo. However, recently new organoid models have been established that could shed light on the initiation, development, tumour invasion, and treatment of glioblastoma. In two independent studies, Bian et al. [115] and Ogawa et al. [116] utilised CRISPR/Cas9 genome editing technology to manipulate cerebral organoids towards tumorigenesis. In both studies, cells derived from the generated tumour organoids exhibited epithelial-mesenchymal properties, indicative that they are representative of the invasive mesenchymal subtype of glioblastoma. Indeed, the cells were invasive when seeded with normal cerebral organoids [115,116] and were capable of forming tumours when xeno-transplanted into mouse recipients [116]. While neither group determined radiation responses of the glioblastoma organoids, Bian et al. demonstrated that CRISPR/Cas9 generated glioblastoma organoid models are appropriate for preclinical in vitro drug screening [115].

Indeed, studies which have investigated the radiosensitivity of glioblastoma organoids have demonstrated that they more closely resemble in vivo tumour sensitivity than monolayer cultures [102,103]. Furthermore, importantly, particularly from a radiobiology point-of-view, Hubert et al. showed that although the non-stem cells of the organoids were radiosensitive, the tumour-initiating cancer stem cells were indeed resistant [102], recapitulating important in vivo findings from previous studies [114]. Although these glioblastoma models offer excellent platforms to study glioblastoma development and biology, and to test new treatments, the duration of culturing generally does not facilitate rapid screening for a more personalised approach to treatment. However, recently a robust and rapid (within 1-2 weeks) protocol for establishing glioblastoma organoids capable of facilitating moderate to high throughput screening for a potentially more personalised response prediction [117].

## 5. The Future Directions of Organoid Models in Radiation Biology

Despite a growing role for organoids in radiobiology (as well as other fields of biology) and continuous advances of the models to faithfully simulate the tissue of origin, organoids still have limitations. However, these drawbacks may represent opportunities. Opportunities for researchers to optimise and improve current organoid systems, and opportunities to complement their research with other techniques, such as clinical imaging techniques for enhancing patient treatment response predictions.

While organoids consist of heterogeneous cell types and are cultured in three dimensions, they still lack important microenvironmental cues, such as sympathetic and parasympathetic innervation and immune cells (such as macrophages and cytokines). These are crucial factors in both development and regeneration of tissue. There is growing evidence for the role of parasympathetic innervation in salivary gland development [118] and regeneration [119], including following radiation-induced damage [120]. Finding means to accurately mimic autonomic innervation in organoids may be important to fully utilising them as models for regeneration in tissues with a similar architecture to the salivary glands. Similarly, the lack of stroma and immune cells in organoids in response to both injury and treatment are important factors which still need addressing especially considering the rising number of applications of immunotherapy. In the aforementioned study of Ooft et al. [92], while the patient-derived organoids were predictive of patient response to irinotecan-based treatments, they were not predictive of 5-FU–oxaliplatin combination therapy, which the authors suggest may, at least in part, be done to the lack of crucial stroma and immune system interactions. Recent advances have been made to overcome these issues, with Neal et al. [121] successfully developing patient-derived organoids with the T-cell spectra of the original tumours capable of modelling the immune checkpoint blockade. Alternatively, co-culturing of organoids with immune cells will offer a theoretically more realistic tissue response. Indeed, in co-culture experiments with macrophages and mammary organoids, macrophages were shown to migrate to organoids with an increased migration rate towards irradiated organoids [122].

Furthermore, stroma also plays an important role in radiation responses, of both normal and tumour tissue. In organoid cultures derived from whole tissue biopsies (without stem cell selection) stromal cells and effects can be found within the culture system [123]. However, in organoid cultures from selected stem cells stroma is absent, and therefore stromal co-culturing is necessary to recapitulate the effects of the tissue’s stroma. In prostate organoids, an increased viability and maintained branching was induced upon co-culture with prostate stroma [124]. Furthermore, the generation of organoids derived from prostate cancer was also improved upon stromal co-culture. These effects were suggested to be primarily due to direct contact with stromal cells and the expression of factors, such as TGF-β, by the stromal cells [124]. Besides the advance that this model represents in development and disease studies, the co-culture of organoids with tissue-specific stromal cells could have important implications for treatment responses, due to the important role of stromal cells [125] and the effects of signalling factors, such as TGF-β [126], in tissue responses.

Radiation-induced bystander effects have been suggested to act both proximally [127,128] and distally [129,130] to the site of irradiation; however, organoids derived from a single tissue currently do not recapitulate such interactions. Various anti-cancer therapies, including radiation, are known to induce senescence and an induction of a senescent associated secretory phenotype [131,132] which, it has been suggested, can in turn contribute to therapy-induced normal tissue side effects [133]. Studies using cultured media from irradiated cells has long been shown to induce paracrine bystander effects in non-irradiated cells [134] and such techniques may be insightful into the effects of secreted SASP proteins on untreated cells or organoids. Indeed, our group recently demonstrated that cultured media from irradiation-induced senescent organoids inhibits organoid forming efficiency in freshly passaged salivary gland-derived organoids [135]; however, these models still lack a true interaction between treated and non-treated organoids and the potential paracrine effects of other tissues in their vicinity in vivo.

Furthermore, both organ–organ, tumour–organ and vasculature interactions are generally absent in organoid cultures. Some of the glioblastoma organoid studies mentioned above elegantly show that cancer cells and healthy cells can be cultured together as organoids allowing for the study of tumour invasion [103,115,116]. Moreover, these models may be useful in revealing new therapeutic targets for tumour radiosensitisation or normal tissue radioprotection. Implementing organoid models alongside newly-established microfluidic devices which allow for the study of metabolic gradients [136] in radiation studies has the potential to reveal valuable insights of how such as signalling gradients can influence both irradiated and non-irradiated cells in perhaps a physiological relevant setting than organoids alone. Indeed, gut-on-a-chip models have recently been utilised in studies of radiation-induced intestinal injury and faithfully mimicked epithelial cell loss due to reactive oxygen production as seen in vivo [137] and may represent an excellent model for complementary studies to the abovementioned “mini-gut” organoid models in radiation studies. Organ-on-a-chip devices have been established for various other tissues, including lung [138], kidney [139] and liver [140]. The capacity of these platforms to mimic functional mechanics, such as breathing movements in lung-on-a-chip [138], could potentially offer more physiologically relevant models to complement and add a translational element to findings from organoid radiation studies. Furthermore, multiple chamber “on-a-chip” devices [141] could overcome the limitation of organoids of studying organs in isolation, in which each chamber potentially could contain cells from different tissues, vessels, stroma or nerves.

Radiation-induced endothelial cell loss and vascular damage are known to be major contributors to the response of both normal tissue and tumours [142,143]. Vasculopathy significantly increases the chances of ischemic stroke following radiation treatment [144], while preclinical models have been used to demonstrate that vascular remodelling is a major contributor to radiation-induced lung toxicity [145]. The vasculature of a tissue is essential for nutrient availability and regeneration following damage, as well as effective engraftment after tissue transplantation [146]. Furthermore, the response of tumour vasculature, particularly vasculogenesis, has also been shown to play a key role in tumour recurrence following radiation treatment [147]. While radiation can initially control the tumour, a reduced flow through tumour blood vessels and increased hypoxia can induce the hypoxia inducible factor-1 pathway. This in turn can activate pathways to re-promote vasculature and can subsequently cause tumour regrowth [143]. Therefore, it is important that in vitro models, particularly tumour models, can recapitulate such vasculature features. Recently, many techniques have been established to engineer vascularisation of organoids, including bioprinting, implantation into highly vascularised tissue and growing organoids in the vicinity of endothelial cell monolayers [148]. Vascularised organoids, such as recently-established vascularised cortical organoids [149,150] and tumour organoids [151], offer new opportunities to study disease pathology but also to study the impact vascularisation can have on treatment (including radiation) responses.

Finally, although there are many different protocols and technical considerations for the isolation and propagation of organoids, they are often arduous and time consuming. In order to have enough cells or organoids to test still often requires weeks to months of culturing. This is of particular importance for the development of organoids as a model for predicting patient responses in proposed precision therapies, where it is frequently necessary to treat patients as soon as possible. Protocols are being established to reduce culture time of organoids while maintaining fidelity of the systems of various different tissue origins (such as the aforementioned glioblastoma organoid model [117]); however, it is important that organoid models are further optimised for rapid and accurate screening of responses before implementation in a personalised medicine.

Despite their limitations, the future of organoid models in the field of radiobiology remains bright. As highlighted, many valuable studies are already overcoming the shortcomings of organoids, and as our knowledge and availability of organoid models grow, so too will their place in radiobiology. New organoid models can potentially shed some much-needed light on tissues which are perhaps less studied or highly limiting to the clinical application of radiation treatment, such as the liver. Moreover, while it could be questioned if a response prediction accuracy of approximately 80–85% is good enough, this will surely only improve as the models themselves are further optimised. Combining clinical patient imaging techniques currently used to predict patient responses, such as PET/CT, with the in vitro predictions from organoids may in the future bring around more accurate means to forecast treatment outcomes. Organoids could also potentially be used in discovery and validation of radiation biomarker and in radiomics. Understanding the mechanisms behind tissue regeneration are key to mitigating radiation-induced side effects, whether it is by stem cell therapy or through druggable targets to protect against damage, and organoids have already proven themselves as excellent models for such studies.

## Figures and Tables

**Table 1:** Radiation response studies using different cancer organoid models.

Tumour Type	Organoids Radiation Treatment	Key Findings	Ref.
Rectal cancer	5-Fluorouracil (5-FU), FOLFOX (5-FU, leucovorin and oxaliplatin) or radiation	Tumour organoids displayed clinically relevant chemo- and radiation responses. Established an orthotopic endoluminal rectal cancer mouse model which reflected patient-specific responses.	[93]
Rectal cancer	Irradiation, 5-FU, or Irinotecan	Colorectal cancer organoids could predict patient outcome in 68 out of 80 patients, based on at least on organoid treatment course.	[94]
Multiple cancers, including lung, colorectal and pancreatic adenocarinomas	5-FU and/or radiation	Colorectal cancer patient-derived organoids displayed differential responses to 5-FU chemotherapy and/or radiation. Prospectively predicted treatment outcome of patient with metastatic colon cancer.	[100]
Head and neck squamous cell carcinoma	Doses ranging from 0–10 Gy	Differential responses which could potentially indicate clinical correlations. However, no resistance mechanisms could be identified via differential gene expression patterns.	[101]
Glioblastoma	Radiation (3 Gy)	Edges of organoids displayed increased apoptosis in Sox2- cells. However, Sox2+ cells (considered as the glioblastoma cancer stem cells) showed an increased resistance.	[102]
Cerebral organoid glioma	Radiation (5 or 10 Gy)	Established organoids combining glioblastoma and healthy cerebral tissue (GLICO). Glioblastoma stem cells showed increased radioresistance in GLICOs compared to when cultured in 2D.	[103]
